# Histone deacetylase inhibitor, Trichostatin A induces ubiquitin-dependent cyclin D1 degradation in MCF-7 breast cancer cells

**DOI:** 10.1186/1476-4598-5-8

**Published:** 2006-02-20

**Authors:** John P Alao, Alexandra V Stavropoulou, Eric W-F Lam, R Charles Coombes, David M Vigushin

**Affiliations:** 1 Department of Cancer Medicine, Cancer Cell Biology Section, Imperial College, Hammersmith Hospital, Du Cane Road, London, W12 0NN, UK

## Abstract

**Background:**

Cyclin D1 is an important regulator of G1-S phase cell cycle transition and has been shown to be important for breast cancer development. GSK3β phosphorylates cyclin D1 on Thr-286, resulting in enhanced ubiquitylation, nuclear export and degradation of the cyclin in the cytoplasm. Recent findings suggest that the development of small-molecule cyclin D1 ablative agents is of clinical relevance. We have previously shown that the histone deacetylase inhibitor trichostatin A (TSA) induces the rapid ubiquitin-dependent degradation of cyclin D1 in MCF-7 breast cancer cells prior to repression of cyclin D1 gene (*CCND1*) transcription. TSA treatment also resulted in accumulation of polyubiquitylated GFP-cyclin D1 species and reduced levels of the recombinant protein within the nucleus.

**Results:**

Here we provide further evidence for TSA-induced ubiquitin-dependent degradation of cyclin D1 and demonstrate that GSK3β-mediated nuclear export facilitates this activity. Our observations suggest that TSA treatment results in enhanced cyclin D1 degradation via the GSK3β/CRM1-dependent nuclear export/26S proteasomal degradation pathway in MCF-7 cells.

**Conclusion:**

We have demonstrated that rapid TSA-induced cyclin D1 degradation in MCF-7 cells requires GSK3β-mediated Thr-286 phosphorylation and the ubiquitin-dependent 26S proteasome pathway. Drug induced cyclin D1 repression contributes to the inhibition of breast cancer cell proliferation and can sensitize cells to CDK and Akt inhibitors. In addition, anti-cyclin D1 therapy may be highly specific for treating human breast cancer. The development of potent and effective cyclin D1 ablative agents is therefore of clinical relevance. Our findings suggest that HDAC inhibitors may have therapeutic potential as small-molecule cyclin D1 ablative agents.

## Background

Cyclin D1 is an important regulator of G_1_-S phase cell cycle transition. Active cyclin D1-cyclin dependent kinase 4/6 complexes phosphorylate retinoblastoma protein, resulting in release of sequestered E2F transcription factors and subsequent expression of genes required for progression into S phase [1]. Cyclin D1 accumulation is required for progression through the G_1 _phase of the cell cycle. Interestingly, cyclin D1 degradation at the end of G_1 _phase is also necessary for progression into S phase and failure to degrade cyclin D1 results in G_1 _arrest [2]. Following S phase, cyclin D1 levels again rise steadily if mitogenic stimuli remain present and elevated levels of cyclin D1 are required for continued cell cycling [3]. Regulating the rate of ubiquitin-dependent degradation enables cells to rapidly adjust the level of cyclin D1 protein despite a constant rate of continued synthesis.

Following its discovery, cyclin D1 was localized to the nucleus and its rapid ubiquitin-dependent degradation shown to require phosphorylation at Thr286 by glycogen synthase kinase 3β (GSK3β) [4]. Additional studies led to the proposal of a model in which at the end of the G_1 _phase, GSK3β migrates into the nucleus where it phosphorylates cyclin D1 [5], resulting in ubiquitylation, nuclear export and degradation of the cyclin in the cytoplasm [4]. Cyclin D1 nuclear export is dependent on the CRM1 complex and requires prior phosphorylation of cyclin D1 by GSK3β. Inhibition of CRM1 with leptomycin B, GSK3β inhibition, or T286A mutation inhibits ubiquitin-dependent cyclin D1 degradation [4-6]. Early experiments suggested that GSK3β-dependent phosphorylation is required for cyclin D1 ubiquitylation [7] but cyclin D1 can also be ubiquitylated independently of GSK3β via unknown mechanisms [8].

Recent studies suggest that cyclin D1 regulation at the protein level may be more complex than previously thought. Firstly, a constitutively nuclear splice variant (cyclin D1b) that lacks the C-terminal domain including Thr286 was neither more stable than the wild type cyclin nor accumulated to excessive levels [9]. These observations are surprising for the reasons stated above. Secondly, Guo *et al*. [3] demonstrated that cyclin D1 is degraded throughout the cell cycle although its destruction is enhanced during S phase. The observation that a Green Fluorescent Protein (GFP)-tagged cyclin D1 T286A mutant was more stable during S phase, linked phosphorylation at this residue to rapid protein degradation. Thr286 phosphorylation therefore enhances cyclin D1 degradation during S phase. However, GSK3β activity was unchanged throughout the cell cycle and the mutant cyclin D1 protein did not accumulate [3]. The observed failure of cyclin D1b or Thr286 mutants to accumulate to excessive levels suggests the existence of an alternative pathway for cyclin D1 destruction that is independent of Thr286 phosphorylation and nuclear export. In addition, the rapid degradation of cyclin D1 by the 26S proteasome following DNA damage does not require either GSK3β activity or Thr286 phosphorylation [10]. Furthermore, Thr286-independent ubiquitylation has been previously described, suggesting that rapid cyclin D1 degradation can occur by other pathways in the absence of GSK3β activity. More recently, the serine/ threonine kinase Mirk/ Dyrk1B was shown to enhance cyclin D1 degradation by phosphorylating Thr288. Mirk activity is restricted to the G0-/early G1-phase of the cell cycle and may not regulate cyclin D1 in actively cycling cells [11].

The Skp2 F-box protein is a specificity conferring subunit of the Skp1-Cullin-F-box (SCF) complex that has been associated with cyclin D1 ubiquitylation [12]. Skp2 'knockdown' by siRNA abolishes degradation and results in accumulation of cyclin D1 [13]. High levels of Skp2 can be detected at S phase, around the time that cyclin D1 phosphorylation and degradation are enhanced [14]. Although much attention has been focused on Thr286 phosphorylation, the decline in cyclin D1 levels during S phase may also reflect increased ubiquitylation and degradation. We have previously shown that the histone deacetylase (HDAC) inhibitor trichostatin A (TSA) induces the rapid ubiquitin-dependent degradation of cyclin D1 in MCF-7 breast cancer cells prior to repression of cyclin D1 gene (*CCND1*) transcription [15] TSA-induced cyclin D1 degradation is associated with Skp2 upregulation and Skp2 siRNA inhibits this response. In MCF-7 cells transiently expressing GFP-cyclin D1, TSA treatment resulted in accumulation of polyubiquitylated GFP-cyclin D1 species and reduced levels of the recombinant protein within the nucleus. TSA therefore induces the rapid loss of cyclin D1 in MCF-7 cells by enhancing its ubiquitin-dependent degradation.

Cyclin D1 ablation has been shown to provide specific protection against breast cancer and can overcome drug resistance by sensitizing these cells to apoptotic signals [16,17]. Anti-cyclin D1 therapy may be thus be important for treating human breast cancer [18,19]. Several HDAC inhibitors with structural similarity to TSA are currently in development or early phase clinical investigation [20-23]. We thus wished to investigate further, the mechanisms underlying the effect of TSA on cyclin D1 degradation in MCF-7 breast cancer cells. Cyclin D1 levels are elevated in this cell line as a result of its defective ubiquitin-dependent degradation [12]. Here we provide further evidence for TSA-induced ubiquitin-dependent degradation of cyclin D1 and demonstrate that GSK3β-mediated nuclear export facilitates this activity. The development of HDAC inhibitors as small-molecule cyclin D1 ablative agents may thus be of clinical relevance.

## Results

### TSA induces cyclin D1 26S proteasomal degradation

**Figure 1:**
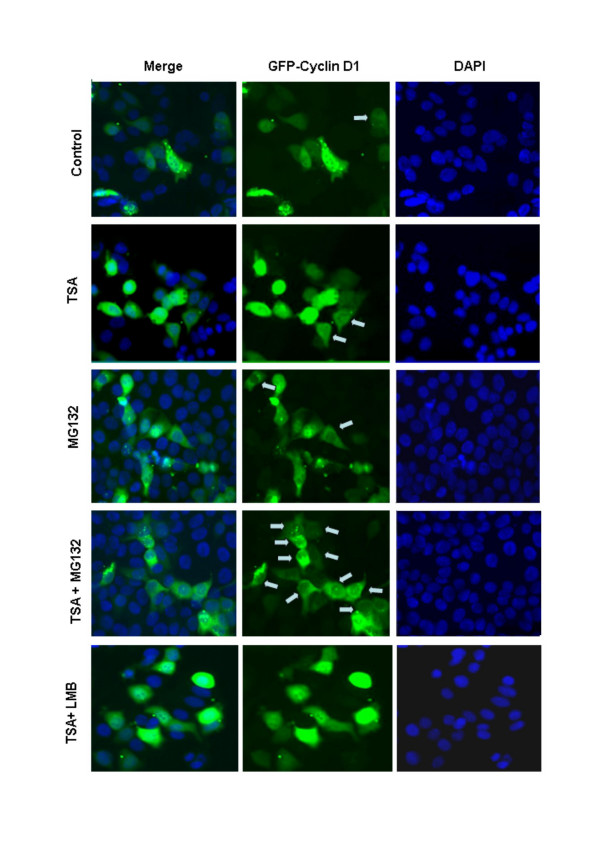
Effect of TSA on GFP-Cyclin D1 localization in MCF-7 cells. Cells were transfected with expression vectors encoding wild type GFP-Cyclin D1 (GFP-Cyclin D1_WT_). 24 h after transfection, cells were treated with TSA (1 μM) alone or in the presence of MG132 (50 μM) or leptomycin B (LMB) (10 ng /ml) for 6 h. Cells were fixed in ice cold methanol for 5 min, counterstained with DAPI and examined by fluorescence microscopy. Cells with predominantly cytoplasmic GFP-cyclin D1 are indicated by grey arrows.

**Figure 2:**
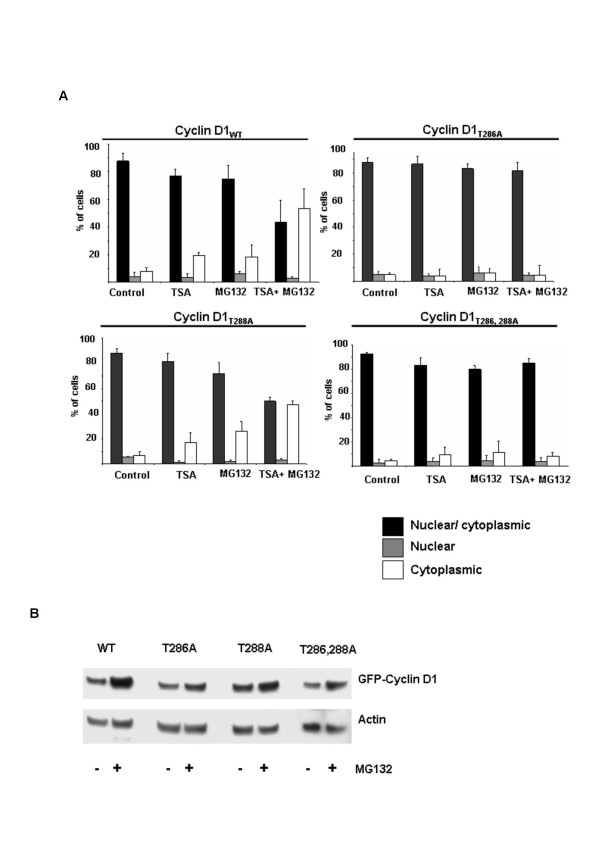
Thr286 phosphorylation mediates the effect of TSA on GFP-cyclin D1 cytoplasmic accumulation A, Thr-286 but not Thr-288 is required for TSA induced cyclin D1 nuclear exclusion. MCF-7 cells were transfected with vectors encoding GFP-Cyclin D1_WT_, GFP-Cyclin D1_T286A_, GFP-Cyclin D1_T288A _or GFP-Cyclin D1_T286,-288A _for 24 h and treated as for Figure 1. GFP-Cyclin D1 localization was scored by quantitative fluorescence microscopy counting >200 cells in three separate experiments. B, Effect of MG132 on wild type and mutant GFP-Cyclin D1 protein levels. Cells were transfected as in A and then treated with MG132 (50 μM) for 6 h. Cell lysates were separated by 4–20 % SDS-PAGE and immunoblot analysis was done using antibodies against GFP and actin.

In previous studies we demonstrated that TSA induces the rapid degradation of cyclin D1in MCF-7 cells. Co-culture of TSA-treated cells with the proteasomal inhibitor MG132 inhibited cyclin D1 degradation, demonstrating a role for the ubiquitin-dependent degradation pathway [15]. In MCF-7 cells transiently expressing GFP-cyclin D1, the recombinant protein localizes to both the cytoplasm and nucleus of most cells (Figure 1, top panel). Co-culture with TSA and MG132 however, promoted the localization of GFP-cyclin D1 within the cytoplasm of most cells. (Figure 1, gray arrows). In contrast, co-culture of MCF-7 cells with TSA and leptomycin B (LMB), an inhibitor of CRM1-dependent nuclear export, promoted the nuclear accumulation of GFP-cyclin D1 (Figure 1, bottom panel). We wished to determine the role of GSK3β in mediating this effect of TSA on cyclin D1 localization. Site-directed mutagenesis was used to mutate wild type GFP-cyclin D1 residues Thr286 and/or Thr288 to alanine. MCF-7 cells transiently expressing wild type or mutant GFP-cyclin D1 were co-cultured with TSA and MG132 for 6 h and subsequently examined by quantitative fluorescence microscopy. Although the expression of the recombinant protein was very high in some cells, a distinct cytoplasmic, nuclear or nucleo-cytoplasmic localization could be observed in most transfected cells (Figure 1). GFP-cyclin D1 localization did not appear to be dependent on the level of recombinant protein expression (Figure 1). Cells with a >80 % cytoplasmic or nuclear localization of the recombinant protein were scored as cytoplasmic and nuclear respectively. All other cells were scored as nucleo-cytoplasmic. More than 200 cells from at least eight separate fields were scored from three separate experiments for quantitation. Wild type and mutant GFP-cyclin D1 localization was predominantly nucleo-cytoplasmic in the majority of cells examined (Figure 1 (gray arrows), Figure 2A). Treatment with either TSA or MG132 alone did not affect GFP-cyclin D1 localization. Co-culture with TSA and MG132 however, resulted in a significant increase in the number of cells in which GFP-cyclin D1 localization was predominantly cytoplasmic. Mutation of Thr286 alone or together with Thr288 abolished the effect of TSA on GFP-cyclin D1 localization. No effect on localization was observed when cells expressing GFP-cyclin D1_T288A _were co-cultured with TSA and MG132. While these experiments did not allow us to distinguish clearly between increased nuclear degradation, nuclear export and cytoplasmic sequestration, they none the less suggested a possible role for GSK3β-dependent Thr286 phosphorylation in mediating the effect of TSA on GFP-cyclin D1 localization. We previously reported, that TSA enhances the accumulation of polyubiquitylated GFP-cyclin D1 when 26S proteasomal degradation is inhibited by MG132 [15]. It is possible that TSA treatment results in the cytoplasmic accumulation of polyubiquitylated cyclin D1 when 26S proteasomal degradation is inhibited.

### GSK3β-dependent nuclear export facilitates the Rapid TSA-induced degradation of Cyclin D1

**Figure 3:**
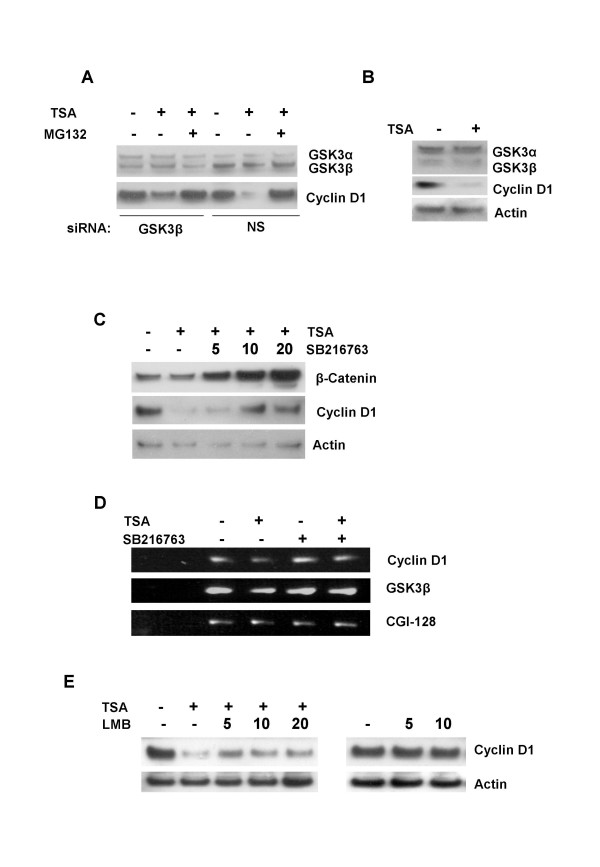
Effect of GSK3β and CRM1 inhibition on TSA induced cyclin D1 degradation. A, MCF-7 cells were transfected with or without GSK3β specific siRNA pools. 72 h after transfection, cells were treated with TSA (1 μM) alone or in the presence of MG132 (50 μM) for 6 h. Cell lysates were separated by 4–20 % SDS-PAGE and immunoblot analysis was done using antibodies against GSK3 α/β, cyclin D1 and actin. B, Effect of TSA on GSK3β levels and activity. MCF-7 cells were cultured in the absence or presence of TSA for 6 h. Immunoblot analysis was done with antibodies against GSK3, cyclin D1 and actin. C, partial inhibition of TSA induced cyclin D1 degradation by the GSK3 inhibitor SB216763. Cells were cultured for 24 h with the indicated concentrations (μM) of SB216763 and then treated for 6 h with TSA (1 μM). Immunoblot analysis was done with antibodies against β-Catenin, cyclin D1 and actin. D, Effect of SB216763 on GSK3β mRNA expression. MCF-7 cells were cultured for 24 h in the absence or presence of SB216763 (10 μM) and treated with TSA (1 μM) 12 h. Semi-quantitative RT-PCR analysis was done with primers for GSK3β, cyclin D1 and CGI-128 as a loading control. E, effect of the CRM1-dependent nuclear export inhibitor Leptomycin B (LMB) on TSA induced cyclin D1 degradation. MCF-7 cells were pretreated for 1 h with the indicated concentrations (ng/mL) of LMB and then treated with TSA (1 μM) for 6 h. Alternatively MCF-7 cells were cultured in the presence of the indicated concentrations (ng/mL) of LMB for 6 h. Immunoblot analysis was done with antibodies against cyclin D1 and actin.

Our previous observation that residue Thr286 was required for the apparent TSA-induced effect on GFP-cyclin D1 localization (Figure 1 and Figure 2A), suggested a role for GSK3β in facilitating TSA-induced cyclin D1 degradation in MCF-7 cells. We therefore investigated the effect of inhibiting GSK3β activity or CRM1-dependent nuclear export on the cyclin D1 degradation response to TSA. Endogenous levels of GSK3β were reduced by siRNA to >70% of the level in mock transfected MCF-7 cells (Figure 3A). GSK3α levels were unchanged confirming the specificity of the siRNA oligonucleotide pool. The cellular levels of cyclin D1 mRNA and protein in GSK3β knockdown cells were comparable to those in mock transfected cells. TSA-induced cyclin D1 degradation was partially inhibited in GSK3β knockdown cells. In contrast, MG132 completely abolished the cyclin D1 degradation response to TSA. Total GSK3α and GSK3β levels were unaffected by TSA, even after 24 h of treatment (Figure 3B). Specific inhibition of GSK3 activity with SB216763 also resulted in only a partial abrogation of TSA-induced cyclin D1 degradation. SB216763 activity was monitored by immunoblot analysis of the cellular levels of β-catenin, an endogenous GSK3β substrate protein. Although pretreatment of MCF-7 cells with up to 20 μM SB216763 for 24 h failed to completely abolish TSA-induced cyclin D1 degradation, β-catenin stabilization was observed at concentrations as low as 5 μM (Figure 3C). The inability of SB216763 to abrogate TSA-induced cyclin D1 depletion did not therefore result from a loss of activity or inappropriately low concentration of inhibitor. These observations suggested that while GSK3β activity could enhance TSA-induced cyclin D1 degradation in MCF-7 cells, this activity was not indispensable for cyclin D1 ubiquitin-dependent degradation. TSA induces cyclin D1 degradation in MCF-7 cells prior to repressing its transcription and without inhibiting protein synthesis [15] The stabilization of β-catenin and partial inhibition of TSA-induced cyclin D1 degradation by SB216763 may be due to the existence of basal GSK3β activity in the asynchronously growing MCF-7 cells used in this study.

We also investigated further, the effect of inhibiting CRM1-dependent nuclear export on TSA-induced cyclin D1 degradation. Treatment of MCF-7 cells with 5–10 ng / ml of leptomycin B alone for 6 h, did not lead to a significant increase in total cyclin D1 levels above those of vehicle control treated cells (Figure 3E). Nonetheless, pretreatment of MCF-7 cells with leptomycin B partially inhibited TSA-induced cyclin D1 degradation and the effect was maximal at 5 ng/ ml (Figure 3E). Inhibition of GSK3β did not result in changes in the level of cyclin D1 mRNA (Figure 3D). We noted that mutation of Thr286 to alanine (Figure 2B), "knockdown" of GSK3 by siRNA or nuclear export with leptomycin B (Figures 3A and 3E), all failed to induce significant cyclin D1 accumulation. Nonetheless, the ability of SB216763 and leptomycin B to partially inhibit TSA-induced cyclin D1 degradation demonstrates the active involvement of GSK3β-mediated nuclear export. These observations suggest that GSK3β-mediated nuclear export enhances the rate of TSA-induced cyclin D1 degradation in MCF-7 cells. The GSK3β-independent degradation of cyclin D1 has been described previously [8]. In addition, we have demonstrated that the inhibition of 26S proteasomal degradation by MG132 results in the accumulation of both wild type and Thr286 mutant GFP-Cyclin D1 in MCF-7 cells (Figure 2B). It is thus possible that TSA can induce cyclin D1 degradation independently of GSK3β in this cell line.

### Inhibition of TSA induced cyclin D1 degradation

**Figure 4:**
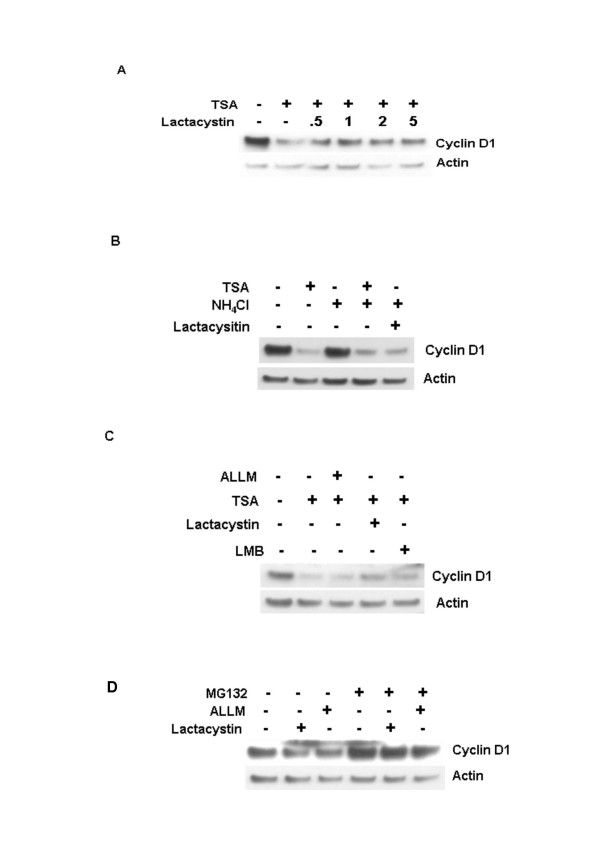
Effect of lactacystin on TSA induced cyclin D1 degradation. A, MCF-7 cells were pretreated with lactacystin (10 μM) for 1 h and then treated with TSA (1 μM) for 6 h. Cell lysates were separated by 4–20 % SDS-PAGE and immunoblot analysis was done using antibodies against cyclin D1 and actin. B, effect of ammonium chloride and lactacystin on TSA induced cyclin D1 degradation. MCF-7 cells were pretreated with the indicated concentrations of ammonium chloride (10 mM) and/or lactacystin (10 μM) for 1 h and then treated with TSA (1 μM) for 6 h. Immunoblot analysis was done with antibodies against cyclin D1 and actin. C, Effects of ALLM, lactacystin and LMB on TSA induced cyclin D1 degradation. MCF-7 cells were pretreated with ALLM (100 μM), lactacystin (10 μM) or LMB (10 ng/mL) for 1 h and then treated with TSA (1 μM) for 6 h. Immunoblot analysis was done with antibodies against cyclin D1 and actin. D, Lactacystin and ALLM induced cyclin D1 loss is inhibited by MG132. MCF-7 cells were treated with lactacystin (10 μM) or ALLM (100 μM) with or without MG132 (50 μM) for 6 h. Immunoblot analysis was done with antibodies against cyclin D1 and actin.

The lack of specificity inherent to many inhibitors has previously led to proteins being linked mistakenly to one degradation pathway or another. Indeed, the peptide aldehydes ALLN and MG132 have been shown to inhibit cysteine and serine proteases such as calpains and cathepsins [24]. We thus investigated the possibility that the stabilization of cyclin D1 in MCF-7 cells by MG132 did not result from the inhibition of 26S proteasome activity alone. Lactacystin, a more specific inhibitor of the 26S proteasome than MG132 [25,26], was used to investigate the possibility that cyclin D1 degradation occurs in the absence of 26S proteasome activity. Lactacystin partially abolished TSA-induced cyclin D1 degradation (Figure 4A). The partial inhibition of TSA-induced cyclin D1 degradation by lactacystin was observable at concentrations as low as 0.5 μM and did not increase even at concentrations as high as 10 μM (Figures 4A,B and 4C). In contrast, inhibition of calpain 1 and calpain 2 by ALLM (Figure 3C) or the inhibition of lysosomal degradation with NH_4_Cl (Figure 4B) or cathepsin inhibitor-1 (CATI-1) (not shown) had no effect on TSA induced loss of cyclin D1 protein expression in MCF-7 cells. It remains unclear why lactacystin is less effective than MG132 at inhibiting TSA-induced cyclin D1 degradation. This differential effect on stability was also observed when cyclin D1 was ablated by treatment with cycloheximide. Similarly, the peptide aldehyde inhibitors ALLN and MG132 but not lactacystin or NLVS (a peptide vinyl sulfone proteasome inhibitor) inhibited cycloheximide-induced cyclin D1 ablation in MCF-7 cells (see Additional file 1). Interestingly, treatment of asynchronous populations of MCF-7 cells with 10 μM lactacystin or 100 μM ALLM for 6 h resulted in decreased cyclin D1 levels (Fig. 4D). Co-treatment with MG132 inhibited lactacystin- or ALLM-induced cyclin D1 loss (Fig. 4D). The inhibition of TSA-induced cyclin D1 degradation by both MG132 and lactacystin confirms our previous observation, that the 26S proteasome plays an important role in TSA-induced cyclin D1 degradation.

## Discussion

In our previous work we found that TSA-induced cyclin D1 degradation is accompanied by Skp2 up-regulation and this activity can be abolished by Skp2 siRNA [15]. The rapid degradation of cyclin D1 has been linked to its phosphorylation on Thr286 by GSK3β. The rapid decline of cyclin D1 during S phase is required for cell cycle progression and has been linked to CRM1-dependent nuclear export and ubiquitin-dependent degradation of the cyclin within the cytoplasm. CRM1-dependent nuclear export of cyclin D1 requires prior phosphorylation of residue Thr286 by GSK3β. It has therefore been proposed that GSK3β migrates into the nucleus at S phase where the kinase phosphorylates cyclin D1 leading to its rapid nuclear export and degradation within the cytoplasm. Although initial studies suggested that cyclin D1 ubiquitylation requires prior phosphorylation by GSK3β, cyclin D1 ubiquitylation can occur independently of this kinase [8,10]. Consistent with this observation, cyclin D1 levels never rise significantly above that of normal cells even when greatly overexpressed at the mRNA level [2]. In addition, cyclin D1b, a splice variant lacking the C-terminal sequence including Thr286, does not always exhibit enhanced protein stability [9]. Interestingly, antizyme-induced cyclin D1 degradation occurs in spermidine treated cells via the 26S proteasome but does not require the prior polyubiquitylation of the cyclin [27]. It should be noted however, that spermidine induced cyclin D1 degradation occurs at a slower rate than that observed following DNA damage or TSA treatment. The precise mechanisms that govern the regulation of cyclin D1 degradation thus remain unclear.

Our present findings suggest that the rapid TSA-induced degradation of cyclin D1 requires GSK3β activity. The inhibition of GSK3β- or CRM1-dependent nuclear export however, only partially inhibited the cyclin D1 degradation response to TSA. In contrast, MG132 completely abolished this protein loss. The underlying reasons for this differential effect are unclear but may due to GSK3β-independent or nuclear degradation of cyclin D1 [8,9]. TSA-induced cyclin D1 degradation could also be inhibited by other 26S proteasome inhibitors such as lactacystin. In contrast, the inhibition of calpains or cathepsins did not affect TSA-induced cyclin D1 degradation.

Cyclin D1 degradation clearly occurs via the ubiquitin-dependent degradation pathway, and its degradation at S phase must therefore be closely liked to the activity of the E3 complexes that mediate its ubiquitylation. It is conceivable, that the up-regulation of a particular E3 complex or its activity and not an increase in GSK3β-mediated nuclear export that is responsible for the decline of cyclin D1 levels at S phase. Although the Skp2 E3 ligase has been linked to cyclin D1 degradation, the exact nature of this relationship remains unclear [13,28]. In proliferating cells, Skp2 levels become elevated around the time cyclin D1 levels decline at the G_1_/S-phase boundary and Skp2 siRNA abolishes this decline. In addition, 26S proteasomes have been localized to both the cytoplasm and nucleus of mammalian cells [29]. It is thus possible, that simply increasing the rate of cyclin D1 ubiquitylation would result in its increased degradation independent of nuclear export. We have shown previously, that TSA-induced cyclin D1 degradation in asynchronously growing MCF-7 cells is associated with a rise in Skp2 protein levels [15]. It is also possible, that TSA induces the expression of other ubiquitin ligases involved in cyclin D1 degradation. We propose that treatment with TSA results in the increased ubiquitylation and degradation of cyclin D1 in MCF-7 cells. The rate of TSA induced cyclin D1 degradation is further enhanced by the constitutive phosphorylation of Thr286 by GSK3β, allowing for the subsequent nuclear export and degradation of polyubiquitylated cyclin D1 within the cytoplasm.

We noted that lactacystin was less effective than MG132 at inhibiting TSA-induced cyclin D1 degradation. The use of appropriate controls has thus been recommended when employing peptide aldehydes as proteasome inhibitors in cell culture studies [25]. Agents that inhibit calpains and cysteine proteases but not proteasomes can be used to rule out the involvement of these proteins. In addition, the involvement of 26S proteasomes in mediating peptide aldehyde sensitive degradation can be confirmed with more specific proteasome inhibitors e.g. lactacystin and epoxomicin [25]. The existence of additional protein degradation pathways has been suggested previously based on studies of protein degradation in rat skeletal muscle [30]. ALLN but not lactacystin inhibited cyclin D1 loss induced by serum starvation or treatment with agents such as actinomycin D and lovastatin [31]. Moreover, studies by Cotelle *et al*., [32] have provided compelling evidence for the existence of an MG132 sensitive cytosolic protease. Our findings clearly implicate the ubiquitin-dependent degradation pathway in facilitating TSA-induced cyclin D1 degradation. We cannot however distinctly rule out at present, the possibility that TSA-induced cyclin D1 degradation can occur independently of GSK3β or 26S proteasomes.

Cyclin D1 has been shown to play an important role in breast cancer cell proliferation [19]. Furthermore, cyclin D1 overexpression has been shown to confer resistance to antioestrogens in breast cancer cells [33] and has been identified as a negative predictive factor for tamoxifen response [34]. Drug-induced cyclin D1 repression not only inhibits breast cancer cell proliferation but also sensitizes these cells to other agents such as CDK and Akt inhibitors [16]. Cyclin D1 ablation thus presents an important therapeutic strategy for the treatment of human breast cancer. Thiazolidinediones and their derivatives have recently been shown to induce proteasome mediated proteolysis of cyclin D1 in MCF-7 cells [18]. In contrast to TSA however, these compounds induce cyclin D1 degradation independently of GSK3β. Our observation that TSA induces cyclin D1 degradation suggests that HDAC inhibitors may also have therapeutic potential as cyclin D1 ablative agents. The identification of novel HDAC inhibitors with potent *in vivo *activity that induce cyclin D1 degradation is thus desirable and forms an active part of our current research focus.

## Conclusion

In the present study, we have investigated further, the mechanisms whereby TSA induces cyclin D1 degradation on MCF-7 cells. We have demonstrated that TSA enhances cyclin D1 degradation at least in part through the ubiquitin-26S degradation pathway. In addition we have also identified GSK3β as an important mediator of TSA-induced cyclin D1 degradation via this pathway. Drug-induced cyclin D1 ablation protects against breast cancer development, inhibits cancer cell proliferation and thus presents an important therapeutic strategy for treating and/ or preventing breast cancer. The findings presented in this study suggest that HDAC inhibitors may have therapeutic potential as cyclin D1 ablative agents for the treatment of breast cancer.

## Methods

### Reagents

Carbobenzoxy-leucyl-leucyl-leucinal (Z-LLL-CHO, MG132), *N*-acetyl-leucyl-leucyl-norleucinal (LLnL, ALLN, MG101), lactacystin, Z-Phe-Gly-NHO-Bz (Z-FG-NHO-Bz, Cathepsin inhibitor I, CATI-1), NIP-Leu_3_-vinyl sulphone (NLVS) and *N-*acetyl-leucyl-leucyl-methional (ALLM, Calpain Inhibitor II) (Calbiochem, VWR International Ltd., Lutterworth, United Kingdom) were dissolved in DMSO at appropriate concentrations and aliquots stored at -80°C. Stock solutions of TSA in ethanol, aqueous ammonium chloride (NH_4_Cl) and leptomycin B in 70% (v/v) methanol (Sigma-Aldrich; Dorset, United Kingdom) were stored at -20°C. The GSK3-specific inhibitor SB216763 (Tocris Bioscience, Avonmouth, United Kingdom) was dissolved in DMSO and stored at -20°C. Antibodies to cyclin D1, actin, Sp1 and GFP (Santa Cruz Biotechnology, Santa Cruz, CA), and β-Catenin (Transduction Laboratories, BD Biosciences Ltd., Oxford, United Kingdom) were used.

### Cell cultures

MCF-7 cells (American Type Culture Collection, Rockville, MD) were cultured in DMEM supplemented with 10% (v/v) fetal calf serum, 2 mM L-glutamine, 100 units/ml penicillin and 100 μg/ml streptomycin at 37°C in humidified 5% CO_2_.

### Site-directed mutagenesis

The pEGFP-cyclin D1 plasmid vector was mutated to create pEGFP-cyclin D1_T286A_, pEGFP-cyclin D1_T288A _and pEGFP-cyclin D1_T286A, T288A_. Site-directed mutagenesis was performed using a QuikChange^® ^II kit (Stratagene) according to the manufacturer's instructions. Mutation of T286A was achieved using the primer pair forward, 5'-TGGACCTGGCTTGCGCACCCACCGA-3', and reverse, 5'-TCGGTGGGTGCGCAAGCCAGGTCCA-3', and T288A using forward, 5'-TTGCACACCCGCCGACGTGCGGGAC-3', and reverse, 5'-AACGTGTGGGCGGCTGCACGCCCTG-3'. The T286A, T288A double mutant was generated by mutating the GFP-Cyclin D1_T286A _plasmid construct with the T288A primer pair.

### Immunoblot analysis

Cells treated as indicated were harvested in 5 ml of medium, pelleted by centrifugation (1,000 × *g *for 5 min at 4°C), washed twice with ice-cold PBS and lysed in ice-cold HEPES buffer [50 mM HEPES (pH 7.5), 10 mM NaCl, 5 mM MgCl_2_, 1 mM EDTA, 10% (v/v) glycerol, 1% (v/v) Triton X-100 and a cocktail of protease inhibitors] on ice for 30 min. Lysates were clarified by centrifugation (15,000 × *g *for 10 min at 4°C) and the supernatants then either analyzed immediately or stored at -80°C. Equivalent amounts of protein (20–50 μg) from total cell lysates were resolved by SDS-PAGE using precast 4–12% Bis-Tris gradient gels (Invitrogen Ltd., Paisley, United Kingdom) and transferred onto polyvinylidene difluoride (PVDF) membranes (Hybond P; Amersham Biosciences United Kingdom Limited, Little Chalfont, United Kingdom) with a Novex XCell system (Invitrogen). Membranes were blocked overnight at 4°C in blocking buffer [5% (w/v) nonfat dried milk, 150 mM NaCl, 10 mM Tris (pH 8.0) and 0.05% (v/v) Tween 20]. Proteins were detected by incubation with primary antibodies at appropriate dilutions in blocking buffer overnight at 4°C. Blots were then incubated at room temperature with horseradish peroxidase-conjugated secondary antibody. Bands were visualized by enhanced chemiluminescence (Supersignal West Pico; Perbio Science UK Ltd., Cheshire, United Kingdom) followed by exposure to autoradiography film (Kodak BioMax ML-light or MR-1).

### Semi-quantitative RT-PCR analysis

For RT-PCR analyses, cells were washed with PBS buffer and lysed directly in the culture plate using RTL buffer (Oiagen). RNA was extracted using an RNeasy Kit (Qiagen) followed by cDNA synthesis and a 25 cycle PCR reaction using a One-Step RT-PCR kit (Qiagen). The annealing temperatures of the primers for Cyclin D1 amplification (Forward 5'-aacagaagtgcgaggaggag-3'; Reverse 5'-ctggcattttggagaggaag-3') was 55°C. GSK3β (Forward 5'-cagcaaggtgacaacagtgg-3'; Reverse 5'-ggaacatagtccagcaccaga-3') and CGI-128 (Forward 5'-ggaattccagcggtggtggttccgcg-3'; Reverse 5'-ccccccgggagctgggaccaggat-3') amplification the annealing temperatures were 58°C and 68°C respectively. Optimization reactions were carried out to ensure the reactions were within the exponential amplification window. Reaction products were resolved on 2 % agarose gels containing 10 μg/ml ethidium bromide and visualized with a UV transilluminatior.

### siRNA/ Plasmid transfection

MCF-7 cells were transfected with commercially available siRNA oligonucleotide pools (Dharmacon, Perbio) using Oligofectamine transfection reagent (Invitrogen) as previously described [15]. Fugene 6 transfection reagent (Roche Diagnostics Ltd, East Sussex, United Kingdom) was used for DNA plasmid transfection. Asynchronous cell populations at a density of 50–60% in 6-well plates or on coverslips were transfected with 1–2 μg of plasmid DNA, following the formation of lipid-DNA complexes for 20 min at room temperature in Optimem I medium (Invitrogen). Complexes were added directly to cells growing in 2 ml DMEM and incubated for 5 h followed by washing with PBS buffer and addition of fresh DMEM. Cells were normally used in experiments 24 h following transfection and the recombinant proteins detected by immunoblotting, or by fluorescent microscopy as previously described

## Abbreviations

ALLM- *N*-acetyl-leucyl-leucyl-methional, ALLN- *N*-acetyl-leucyl-leucyl-norleucinal, CATI-1- cathepsin inhibitor-1, DMEM- Dulbecco's modified eagle medium, DMSO- dimethyl sulphoxide, GSK3β- glycogen synthase 3 beta, HDAC- histone deacetylase, LLnL, LMB- Leptomycin B, NH_4_Cl- ammonium chloride, NLVS- NIP-Leu_3_-vinyl sulphone, PBS- phosphate buffered saline, RT-PCR- reverse transcription- polymerase chain reaction, TSA- trichostatin A, Z-FG-NHO-Bz- Z-Phe-Gly-NHO-Bz, Z-LLL-CHO- carbobenzoxy-leucyl-leucyl-leucinal,,.

## Competing interests

The author(s) declare that they have no competing interests.

## Authors' contributions

JPA, DMV, EW-FL and RCC conceived of the study, coordinated its design and execution and drafted the manuscript. JPA and AVS carried out site-directed mutagenesis, RT-PCR analyses, immunoblot experiments and immunofluorescence microscopy. JPA, DMV, AVS and EW-FL interpreted and analyzed the data. All authors read and approved the final draft manuscript.

## Supplementary Material

Additional File 1 Inhibition of TSA and cycloheximide-induced cyclin D1 ablation. A, MCF-7 cells were pretreated with lactacystin (0.5–5 μM) for 1 h and then treated with TSA (1 μM) for 6 h. Cell lysates were separated by 4–20 % SDS-PAGE and immunoblot analysis was done using antibodies against cyclin D1 and actin. B, MCF-7 cells were pretreated with lactacystin (0.5–5 μM, top panel) or MG132 (50 μM, bottom panel) for 1 h and then treated with cycloheximide (Chx) (5–50 μM) for 1 h. Cell lysates were analyzed as in A. C, MCF-7 cells were pretreated with lactacystin (10 μM), NLVS (10 μM), ALLN (100 μM) or MG132 (50 μM) for 1 h and then treated with Cycloheximide (50 μM) for 2 h. Cell lysates were separated by 4–20 % SDS-PAGE and immunoblot analysis was done using antibodies against cyclin D1 and actin. Click here for file
