# Supplementary figures and images for: Histone deacetylase inhibitor, Trichostatin A induces ubiquitin-dependent cyclin D1 degradation in MCF-7 breast cancer cells
Source: Mol Cancer. 2006 Feb 20;5:8. doi: 10.1186/1476-4598-5-8 (PMC1397858; doi:10.1186/1476-4598-5-8)

## Slide 1
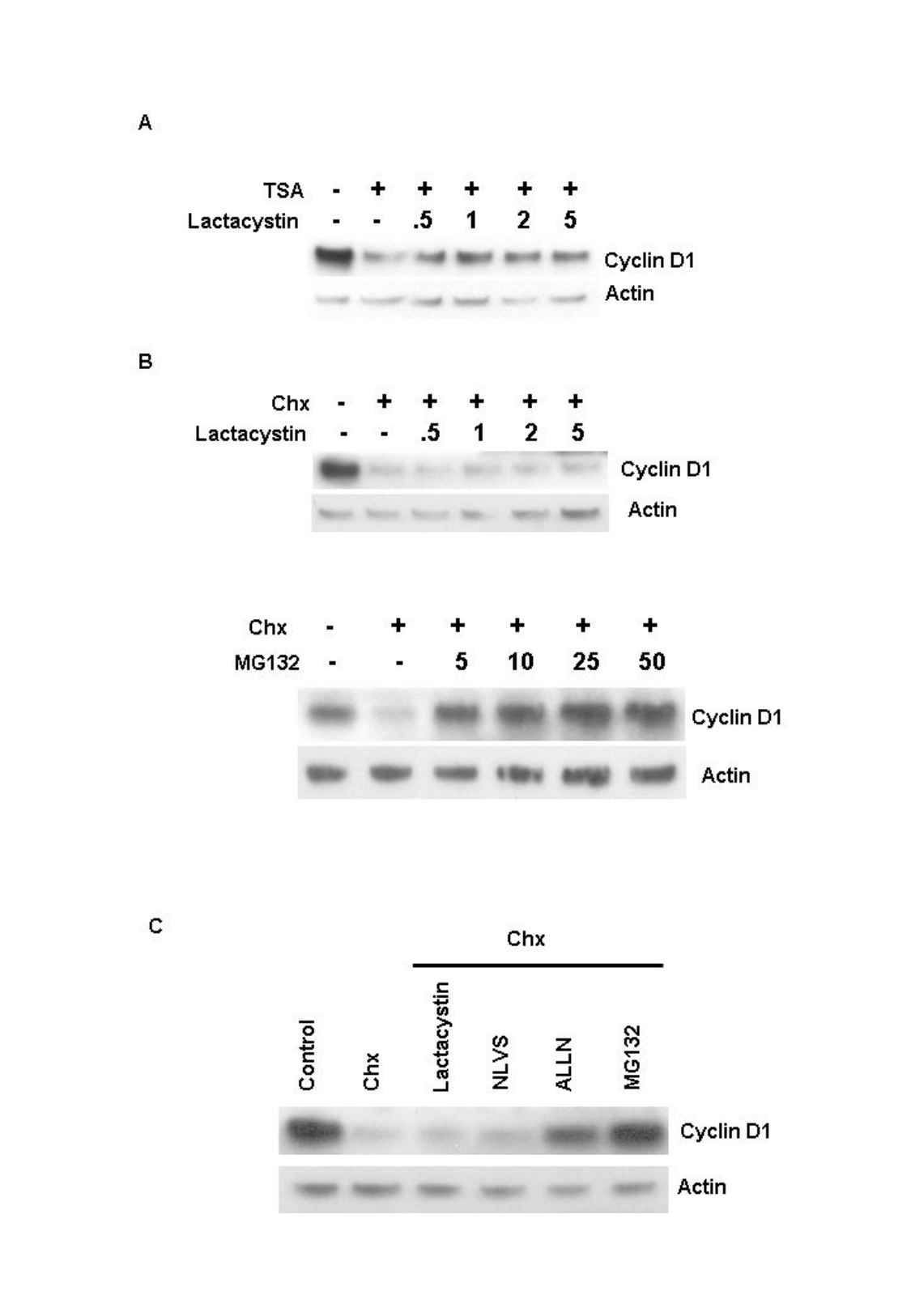

Supplement: Additional File 1 — Inhibition of TSA and cycloheximide-induced cyclin D1 ablation. A, MCF-7 cells were pretreated with lactacystin (0.5–5 μM) for 1 h and then treated with TSA (1 μM) for 6 h. Cell lysates were separated by 4–20 % SDS-PAGE and immunoblot analysis was done using antibodies against cyclin D1 and actin. B, MCF-7 cells were pretreated with lactacystin (0.5–5 μM, top panel) or MG132 (50 μM, bottom panel) for 1 h and then treated with cycloheximide (Chx) (5–50 μM) for 1 h. Cell lysates were analyzed as in A. C, MCF-7 cells were pretreated with lactacystin (10 μM), NLVS (10 μM), ALLN (100 μM) or MG132 (50 μM) for 1 h and then treated with Cycloheximide (50 μM) for 2 h. Cell lysates were separated by 4–20 % SDS-PAGE and immunoblot analysis was done using antibodies against cyclin D1 and actin. [file 1476-4598-5-8-S1.ppt]
